# Ultralow Profile, Low Passive Intermodulation, and Super-Wideband Ceiling Mount Antennas for Cellular and Public Safety Distributed Antenna Systems

**DOI:** 10.3390/s20092456

**Published:** 2020-04-26

**Authors:** Kok Jiunn Ng, Mohammad Tariqul Islam, Adam M. Alevy, Mohd. Fais Mansor

**Affiliations:** 1 Laird Connectivity, Plot 522, Lorong Perusahaan 3, Prai Industrial Estate, Penang 13600, Malaysia; 2 Department of Electrical, Electronic and Systems Engineering, Faculty of Engineering and Built Environment, Universiti Kebangsaan Malaysia (UKM), Selangor 43600, Malaysia; m.mansor@ukm.edu.my; 3 Laird Connectivity, 1 Perimeter Rd #700, Manchester, NH 03103, USA; adam.alevy@lairdconnect.com

**Keywords:** cellular and public safety antenna, in-building antenna, low profile low passive intermodulation, super wideband

## Abstract

This paper presents an ultralow profile, low passive intermodulation (PIM), and super-wideband in-building ceiling mount antenna that covers both the cellular and public safety ultra high frequency (UHF) band for distributed antenna system (DAS) applications. The proposed antenna design utilizes a modified 2-D planar discone design concept that is miniaturized to fit into a small disc-shaped radome. The 2-D planar discone has an elliptical-shaped disc monopole and a bell-shaped ground plane, a stub at the shorting path, with asymmetrical structure and an additional proximity coupling patch to maximize the available electrical path to support the 350 MHz band range. The proposed design maximizes the radome area with a reduction of about 62% compared to similar concept type antennas. Besides, the proposed design exhibits an improved radiation pattern with null reduction compared to a typical dipole/monopole when lies at the horizontal plane. A prototype was manufactured to demonstrate the antenna performance. The VSWR and radiation pattern results agreed with the simulated results. The proposed antenna achieves a band ratio of 28.57:1 while covering a frequency range of 350–10000 MHz. The measured passive intermodulation levels are better than −150 dBc (2 × 20 Watts) for 350, 700 and 1920 MHz bands.

## 1. Introduction

**Figure 1:**
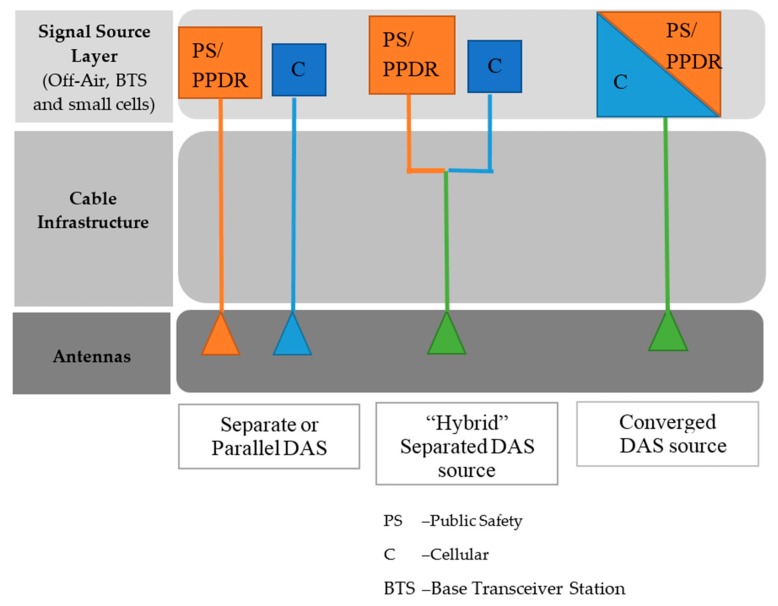
Three enabling DAS approaches for commercial cellular and PS indoor coverage.

The evolution of mobile wireless communication systems has shifted its focus from voice to mobile broadband applications that seamlessly support high data streaming, high quality of experience, ultra-low latency and high throughput anywhere and anytime. The statistics from the industry present that nearly 80% of all mobile usage or traffic happening inside buildings [1,2]. Therefore, the distributed antenna system (DAS) has been regarded as an essential solution to meet the rising demand for indoor coverage and capacity. Recently, efforts have been taking place to include the public safety (PS) or protection and disaster relief (PPDR) telecommunication systems in the long term evolution (LTE) DAS network. Therefore, DAS networks need to support PS/PPDR bands such as very high frequency (VHF) and ultra-high frequency (UHF—including 380, 700 and 800 MHz bands). Figure 1 illustrates the three enabling DAS approaches for commercial cellular and PS/PPDR indoor coverage. 

The following describes the DAS approaches [1]:

- Separate or parallel DAS layers that have discrete infrastructure including cable feeds and antennas for the cellular and PS/PPDR
- “Hybrid” separated DAS layers that have separate cable infrastructure (i.e., PS/PPDR on one fiber from the head-end and cellular on another fiber) but then combine both services on the same antenna
- Commercial cellular combined with PS/PPDR frequency bands on same DAS infrastructure is Converge DAS

With the hybrid or convergent DAS approach, the requirement of a single super wideband antenna to cover both PS/PPDR and cellular has been gaining more attention. It has led to the launch of products by industry. These industrial ceiling mount antennas cover a wide range of bandwidths that include a frequency range of 380–6000 MHz, which supports the typical PS/PPDR UHF 380 MHz band (380–520 MHz range). In several patents [3,4,5,6,7], industries used different monopole shapes (mainly cone-like shaped) as its radiating element over a flat circle ground plane. In [3], a ground leg with multiple stubs from the ground plane was capacitively coupled to the cone radiating element via a lumped capacitor to enhance the bandwidth of the antennas. In [4], a crossed printed circuit board is assembled as the monopole radiating element with a shorting stamping part to the ground plane. The design in [5] used a conical radiating element that parasitically coupled with an annular ring that has a leg shorted to the ground plane. Reference [6] carefully includes the low passive intermodulation (PIM) construction design detail into the design in [5] to improve the PIM level, especially for the 380 MHz band that cannot be achieved by conventional constructions. The cup-type plum-shaped-liked cone in [7] has three shortings, two of them with series resistors. Table 1 shows the known standard sizes of the antennas used in the industry. The antennas are typically high profile, with a typical height of about 150 mm ([8,9]) or 124–133 mm [10,11,12]. 

However, building owners who have high aesthetic expectations may not accept this typical profile height to blend seamlessly with their building ceiling. In recent years, a new class of ultralow profile (ULP) omnidirectional ceiling mount antenna has gained more attention [13]. Considering the thinnest planar antennas using a printed circuit board (PCB) protected with plastic housing, the total height of the ULP antenna can be less than 10 mm. The planar antenna typically consists of a printed slot, dipole, or monopole antenna that are lying horizontally to realize ULP features. The horizontal orientation of these antennas produces a non-omnidirectional radiation pattern in the azimuth plane. In order to achieve an omnidirectional radiation pattern, an array of slots or dipole radiating elements with or without parasitic strips or directors are arranged in the circular configuration as in [14,15,16,17]. However, these designs suffer from limited bandwidth (1.58–3.8 GHz). A tightly arranged circular array of Vivaldi antenna in [18,19] that with a smaller size compared to similar slot array in [17] can attain a better bandwidth with the band ratio up to 9.9:1.

Nevertheless, the achieved band ratio is still insufficient. It has a substantial size if the antenna is scaled to operate from the lowest frequency, *fl* = 350 MHz (the estimated size of ~546 mm Dia. based on 0.64 *λ_fl_* Dia. in [18]). Therefore, single radiating elements with planar dipole types, such as a bow tie and an elliptical or a circular radiating arm remain as suitable candidates for the size factor. Table 2 shows a comparison of lowest profile antennas [20,21,22] in industry, which includes both the PS/PPDR UHF and cellular frequency ranges. However, these antennas still have limitations in terms of frequency range coverage that not able to fulfil the additional mobile LTE band. For example, [20] covers only 380–470 and 1427–2700 MHz with dimensions of 265 × 182 × 15 and [21] covers only 380–960 MHz with the size of 311 (Dia.) × 9.5 mm.

To support both the cellular and PS/PPDR frequency range of 350–6000 MHz, a band ratio of more than 17.14:1 is needed. It is worth noting that any antenna with a ratio bandwidth more than 10:1 is classified as a super wideband antenna [23,24]. Recently, some attractive planar super wideband designs that realize the vertical polarized omnidirectional antennas have been reported. In [23], a bulbous concept dipole of the curve arm with circular-serrated edges exhibits a wide impedance band ratio of 10:1 (300–3000 MHz). Although it covers a wide frequency range, it has long arms with a total length of 453.6 mm. A bowtie radiating elements loaded with loops design [25] can cover a wide frequency range from 420 to 5500 MHz. It has the size of 230 × 230 × 30 mm that with a higher profile due to the two stack of loop structures. The super wideband asymmetrical dipole of circular shape in [26] and elliptical monopole on trapezoid ground plane in [27,28,29,30] offers smaller size compared to the symmetrical antenna. The elliptical monopole antenna resembles a 2D planar discone antenna. However, the size of the scaled design to meet lowest frequency 350 MHz may still be considered significant and inherit a deep null azimuth radiation pattern in most of the frequency ranges when lies at the horizontal plane. The deep nulls may lead to unstable connectivity due to blind spots of coverage. In [13,31], an asymmetrical dipole is with reduced null azimuth radiation pattern compared to a typical dipole but only supports the standard frequency range for long term evolution (LTE) band of 698–3800 MHz. 

In this paper, a novel modified 2D planar discone-like antenna that meets the ULP requirement for indoor DAS is presented. The modified 2D planar discone is based on an asymmetrical elliptical monopole with a bell-shaped ground plane. Various techniques are combined to miniaturize the antenna to fit into a small disc radome that is fed by a pig-tail coaxial cable. The monopole is proximity shorted to the ground plane with a thin path element. A proximity patch on top of the monopole extends the electrical length. The novel antenna covers super wideband with a band ratio of 28.57:1 (a frequency range of 350–10,000 MHz) in a miniaturized size. Besides, the proposed design exhibits reduced null in the azimuth plane compared to a conventional monopole/dipole antenna. Having achieved the low PIM level, the design required a substantial effort to follow the explicit rules in design detail and process in all stages of assemblies. The failure to understand the imperfections and any possible non-linear characteristic properties inherent to the type of material, inter-conductor joint, material finishing, etc. can lead to unstable and low reliability of the PIM level performance. It is worth noting that a regular prototype can demonstrate typical VSWR and radiation pattern performance, but not meet the PIM requirement. As a result, several iterations of high-quality prototypes were produced and experimentally tested for performance verification. 

## 2. Antenna Design and Development

Table 3 summarizes the requirement of a ULP ceiling mount antenna from the specification analysis of the commercial ceiling mount antenna [8,9,10,11,12,20,21,22] that enables PS/PPDR (UHF) and cellular DAS applications. The summary indicates that the standard frequency range is 380–6000 MHz. Still, 350 MHz band is essential for PS/PPDR applications in certain countries or regions (e.g., China). It has been observed that it is challenging to meet the typical requirement of VSWR < 2.1 (especially the 380 MHz band) as some of the industrial products have a VSWR < 2.5 in this band. Typically, the preferred radiation pattern is pure omnidirectional. However, a trade-off in the radiation pattern omnidirectionality arises when the emphasis is shifted for the ULP form factor. Nevertheless, the network coverage planers may still require the antenna to have azimuth reduced null radiation pattern to avoid prominent blind spots at certain angles in the DAS system. 

### 2.1. Simulation Analysis of Conventional 2D Discone-Liked Antenna design

**Figure 2:**
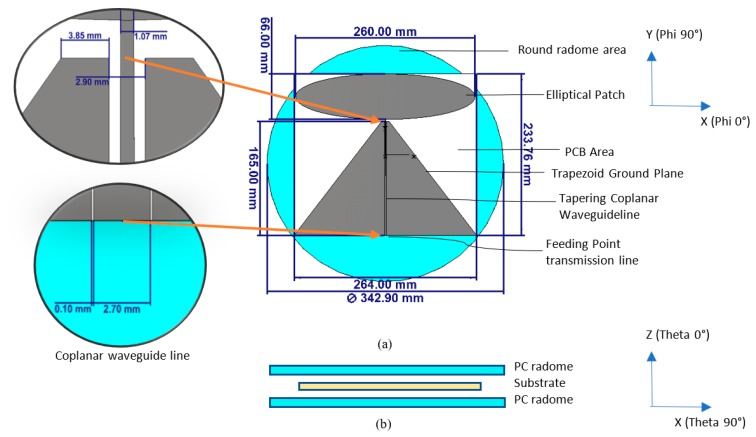
(**a**) Top View and (**b**) Side View of conventional super wideband 2D planar discone (elliptical monopole radiating element with trapezoid ground plane) and tapering coplanar transmission line without adjustment to fit into the small round radome.

Firstly, the required size of the antenna design based on the concepts from [21,23,24] was studied to meet the lowest frequency of 350 MHz through simulation with CST Microwave studio. Figure 2 illustrates an elliptical monopole antenna with a trapezoid ground plane and tapering transmission line. We resized the antenna design to about 264 × 233.76 mm on a AD300 C substrate (*Ɛr* = 2.97 and *tanδ* = 0.0020 at 10 GHz) to meet the lowest frequency down to 350 MHz. This translates that the minimum diameter of a disc-shaped radome is about 342.9 mm (0.5 λ_fl_) without modification. However, this diameter is large. The material used for radome is polycarbonate (PC) with dielectric constant, *Ɛr* = 2.9.

**Figure 3:**
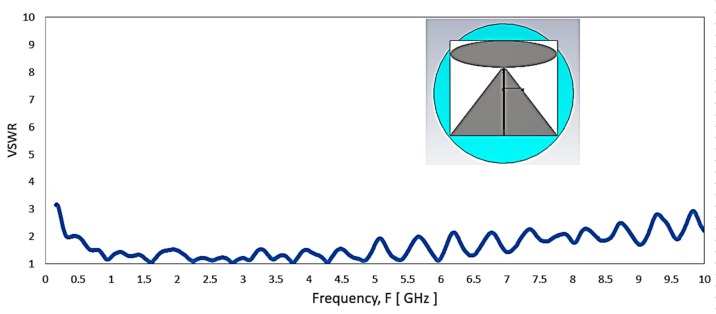
The simulated VSWR plot of the elliptical monopole with trapezoid ground plane that the lowest frequency tweaked to 350 MHz.

**Figure 4:**
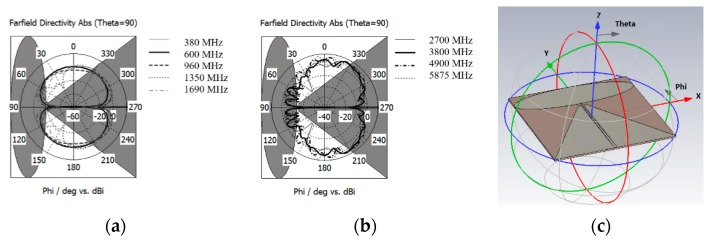
The simulated azimuth radiation pattern (Theta = 90° plane) of the elliptical monopole with trapezoid ground plane lied at the horizontal plane for the frequency range of (**a**) 350–1690 MHz and (**b**) 2700–5875 MHz. (**c**) The coordinate system of the model.

Figure 3 shows the VSWR for the resized antenna design that meets the frequency requirement from 350 MHz with a marginal performance at this lowest frequency band. In such, the antenna was with its minimum size (280 × 233.76 mm) required to reach the lowest frequency for reference. Besides, the frequency range shows the antenna matched well until about 6 GHz. Figure 4 shows the azimuth (theta = 90°) plane radiation pattern of this type of antenna when lies at the horizontal plane. As the design is a monopole type antenna or an asymmetrical dipole, its radiation pattern remains with deep null at Phi = 90° and Phi = 270° consistently across the operating frequency, as shown in Figure 4. The exhibited null at theta = 90° plane (X-Y plane of the antenna) for the ceiling mount antenna is not desirable as it causes the problem of blind spots for its coverage. The following are the modification strategies required for realizing a practical ULP antenna to meet the requirement in Table 3:

- reduced size of radiating element to fit into a smaller diameter radome
- a pigtail cable feed to the antenna from the center of the radome,
- low PIM
- reduced null in azimuth plane radiation pattern
- super-wideband

### 2.2. Design Steps of the Proposed Antenna Design

In the proposed design, a disc-shaped radome with a diameter of 270 mm is used to house the radiating element. This diameter limits the space for the rectangular dimension of the conventional 2D-discone antenna of Figure 2 to about 212 × 180 mm compared to 264 × 233.76 mm (for 342.9 mm dia. radome). It reduces the available area to approximately 62% compared to the 342.9 mm diameter radome. 

**Figure 5:**
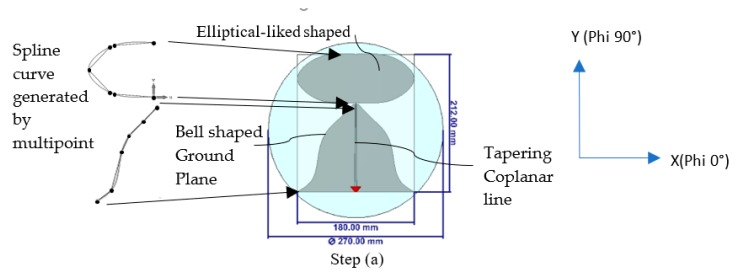

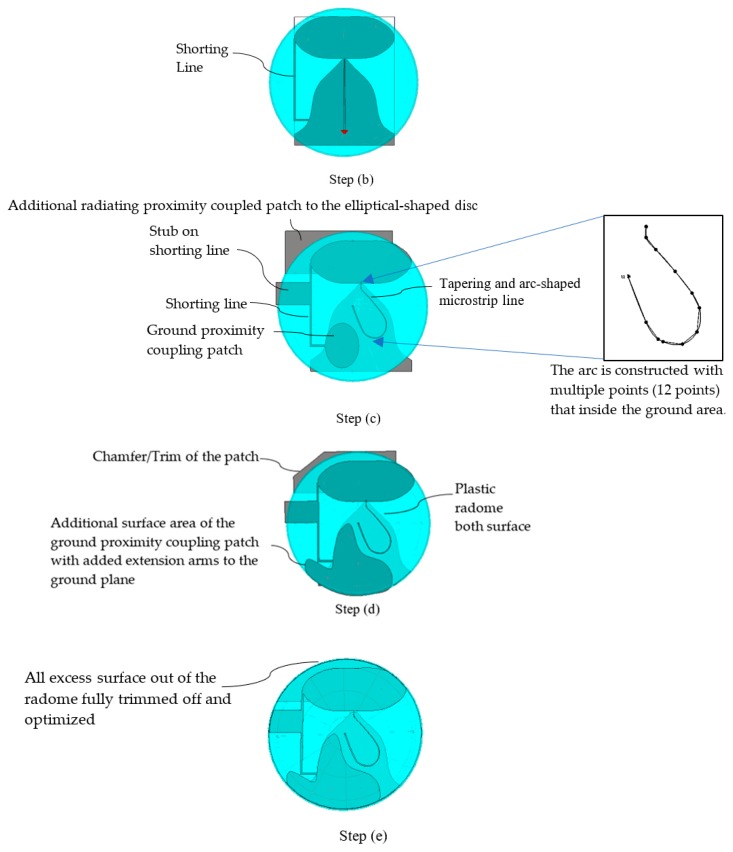
Evolution steps to make the antenna fit into the disc-shaped radome for ceiling mount antenna configuration. Step (**a**) Elliptical-shaped liked monopole with bell-shaped ground plane and tapering coplanar line; Step (**b**) Shorting line added in between the top radiator and the ground plane; Step (**c**) Conversion of the coplanar line to microstrip line, added stub on shorting line and proximity coupling patch (for radiating and ground portion); Step (**d**) Increase proximity ground patch size near the ground shorting line; Step (**e**) trim portion of the patch to fit into the radome and further optimized to have good impedance match at the lowest frequency range.

**Figure 6:**
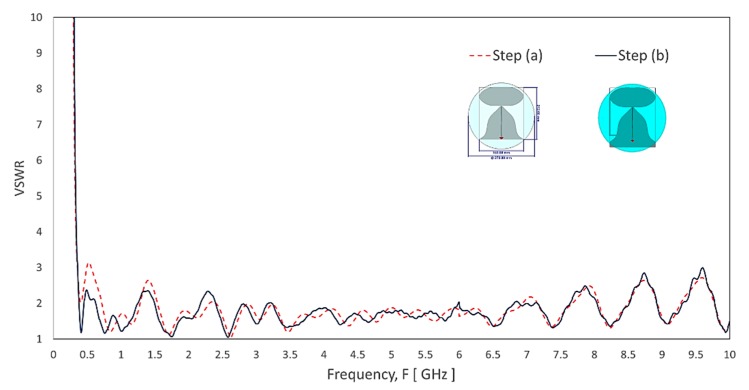
The simulated VSWR of the evolution steps from Figure 5: Step (a) and Step (b).

Figure 5 illustrates the modifications in evolution steps to fit the radiating element into a small disc-shaped radome in about 270 mm dia. Step (a) exhibits the construction of an elliptical-liked shape with multiple splines points instead of the typical curve construction of the x-diameter and y-diameter of elliptical shape. The additional spline curve points allow more freedom to tweak the curvature of the structure, especially those points close to the center between the disc and ground plane for better wideband matching. A few spline points with a bell-shaped structure constructs the ground plane of the antenna instead of the typical trapezoid-shaped ground plane. The transmission line remains as a tapering coplanar line. The inward edge between the ground plane and the elliptical radiating element is essential to ensure a good impedance match over a wide range of frequency. The whole radiating element is confined within the size of 212 × 180 mm by noting that the upper corner area of the rectangle remains unused due to the elliptically shaped radiating element. With this size, the antenna is matched at the lowest frequency of 710 MHz, as shown in Figure 6.

For the antenna to operate from the lowest required frequency of 350 MHz in a small disc-shaped radome, the following describes the three main miniaturization approaches:

- introduce a short between the elliptical-liked shaped radiating element and the ground plane;
- offset the antenna to have it non-symmetrical to any plane to maximize the electrical path;
- grow the structure to substantial any unused space of the radome within the 270 mm diameter.

Figure 5 shows step (b), introducing a shorting line between the top disc radiating element and the ground plane. The shorting line not only reduces the size of the antenna but also plays a vital role in reducing the null of the radiation pattern in a horizontal plane at specific frequency ranges. The shorting location is determined to have less impact on high band performance. In addition to this shorting line, the ground plane length increases by about 20 mm (with some of the trimmed areas) to maximize the available space. As shown in Figure 6, step (b) has the VSWR match from 380–456 MHz with narrow bandwidth. A few frequencies range has slightly higher than 2:1 VSWR from 456 to 2400 MHz and match up to 6.5 GHz for both steps (a) and (b).

**Figure 7:**
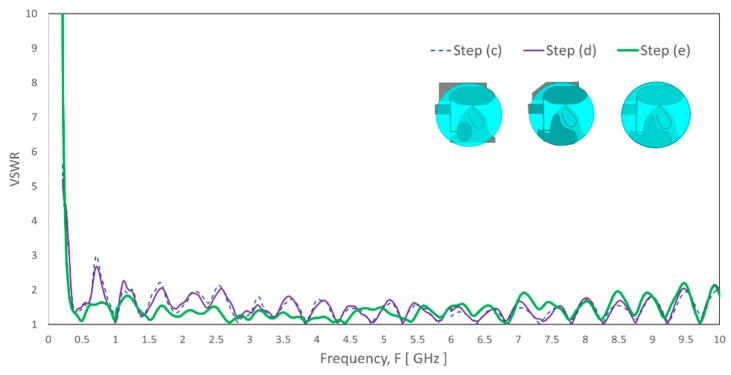
The simulated VSWR of the evolution steps from Figure 5: Step (c) to Step (e).

Figure 5 shows Step (c) of the preparation to feed the radiating element at the center of the antenna with coaxial cable. This preparation includes the following steps:

- Move the top layer bell-shaped ground plane to the bottom layer of the PCB to allow the microstrip line printed on the top layer and enables feeding the coaxial cable perpendicularly to the PCB board with its braid soldered to the ground plane at the bottom layer.
- The microstrip line is maintained at a certain length with its tapering feature as a wideband transformation impedance. With the cable feeding point at the center of the radome, the transmission line curve routes to the center of the antenna. The curl curve is constructed with 12 spline points within the area of the ground plane and manually adjusted for optimization in the CST simulation tool. This feature enables better matching for higher frequency range, as shown in Figure 7.

In step (c), bandwidth at the 350 MHz band enhancement and the lowering the operating frequency approaches includes the following:

- The whole radiating structure offsets to the right hand and to the top to allow the maximization of the available space in the round radome to grow the electrical length of the antenna sufficiently. The outcome that the whole antenna is asymmetrical in both X-Z and Y-Z planes.
- Radiating proximity coupled patch is introduced at the bottom layer of the left-hand side of the top elliptical radiator to maximize space usage and increase the electrical path. This patch is proximity-coupled to the elliptical top radiator with adequate overlapping area coupling and fills up the unused space at the top.
- Also, by introducing a stub along the shorting line, the VSWR improves at the lowest frequency range, where it covers a broader band 342 MHz–642 MHz, as shown in Figure 7.

The shorting element remains at the top layer of the PCB with the radiating elliptical-shaped patch while the ground plane is in a different layer. The conventional approach is using plated through-hole (PTH) via to direct couple the shorting line to the ground plane in a separate layer. The proposed design adds a proximity coupling ground patch at the end of the shorting line above the ground plane. This allows the shorting line to be electromagnetically coupled to the ground plane. PTH may be prone to quality defect risks during fabrication, e.g., not smooth or non-homogeneous surface of the plating. This may lead to a non-linear characteristic that subsequently became the PIM source.

**Figure 8:**
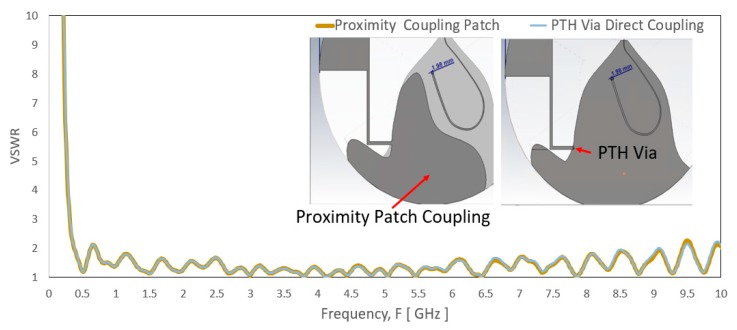
The simulated VSWR between using proximity coupling patch and PTH via direct coupling at the end of the shorting line to the ground plane.

Although step (c) increases the bandwidth for the range of 350 to 520 MHz, the VSWR for a few frequencies higher than 2:1 for frequency below 2700 MHz. The ground coupling patch area to the ground plane is inadequate to have functional coupling to the ground plane that leads to the VSWR spike at 726 MHz. In step (d), a larger surface of the coupling patch slightly improves the VSWR spike at 726 MHz. The ground coupling patch outline partially follows the shape of the ground plane, especially near to the transition between the top radiating element and the ground plane to ensure the impact on the impedance of the high band is minimum. Its outline avoids the nearby transmission line that may couple to the transmission line. Figure 8 shows the comparison of simulated VSWR result between the proximity patch coupling and the PTH direct coupling. As observed, the proximity patch coupling shows overlap curve result that indicates it has a similar coupling effect as the PTH direct coupling. Besides, an extension arm is added into the proximity coupled patch to increase the electrical length of the antenna slightly by expecting that part of the upper patch trimmed where the electrical length reduces.

Step (e) trims excess copper traces in accord to the circumference of the radome to have overall radiating elements fit inside the radome, which reduces the electrical length. This trimming has less impact on the high-frequency range. However, it needs optimization of the shorting stub (*ShortstubW*, *ShortstubL*, *ShortStubP*), parasitic patch (*Patch2L*, *Patch2W*), and the extension arm (*ExtX1, ExtY1*) of the ground coupling patch to meet VSWR < 2:1 at for the lowest frequency range.

### 2.3. The Proposed Antenna Design Geometry

**Figure 9:**
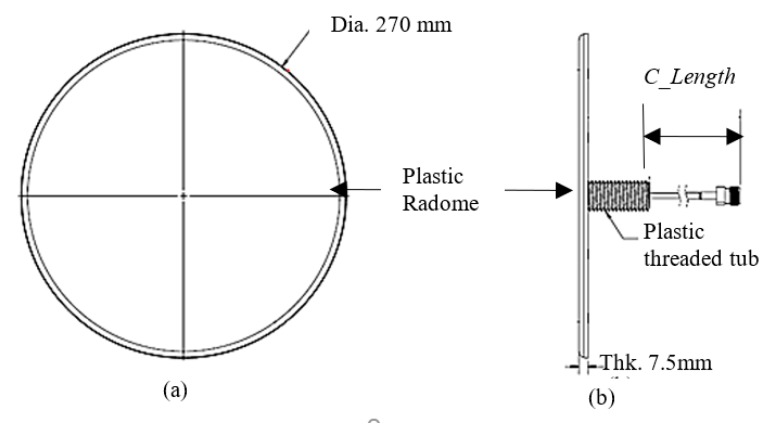
(**a**) Top view and (**b**) side view of the proposed ultralow-profile, ceiling mount antenna’s form factor with pigtail cable.

Figure 9 shows a drawing of the proposed antenna in the PC plastic (*Ɛr* = 2.9) radome with a plastic threaded stub for ceiling mount application. The size of the antenna radome is 270 (dia.) ×7.6 mm. The plastic stud and a nut ease the installation of the antenna to the ceiling board. A formable coaxial cable assembled with a 50 Ohm N-connector or 4.3–10 connector feeds at the center of the antenna. The pigtail cable length, *C_Length* is 300 mm from the plastic stud as the typical cable length in the ceiling mount product. 

**Figure 10:**
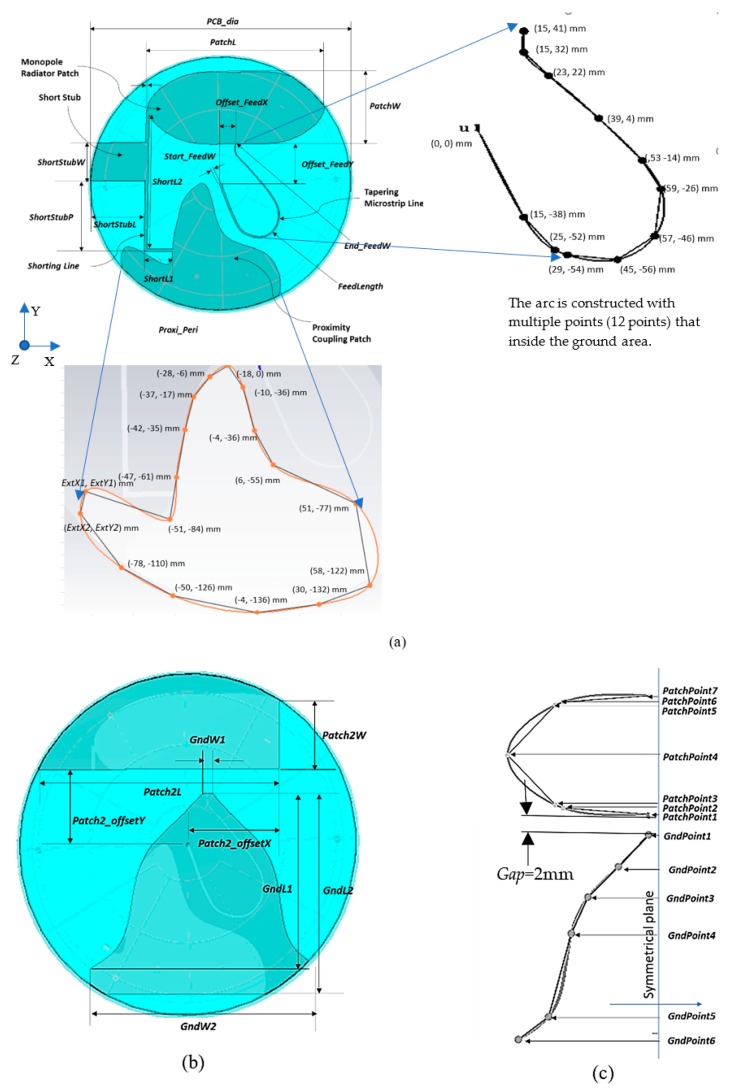
The internal structure of the proposed antenna with a transparent view of the substrate and radome. (**a**) The top layer, (**b**) The bottom layer, and (**c**) the spline points to construct the spline line of the patch and the ground plane.

Figure 10a,b show the internal PCB of the antenna with the top and bottom layers, respectively in a translucent radome. The PCB substrate uses AD300 C (dielectric constant, *Ɛr* = 2.97, and tangent loss, *tan δ* = 0.002 at 10 GHz) from Rogers Corp. (Chandler, Arizona, USA). The top layer consists of a microstrip line, an elliptical-liked shape radiating patch, a ground proximity coupling patch, a shorting line, and stub arm at the shorting line. The microstrip line width is tapering with the starting width of *Start_FeedW,* and *End_FeedW* that curls from the center of the antenna to the offset feeding point (*Offset_FeedX, Offset_FeedY*). The monopole elliptical-liked radiator patch has *PatchL* and *PatchW* that constructed by the multiple spline points, as shown in Figure 10c. Also, the figure shows the spline points that build the bell-shaped ground plane for the bottom layer. The spline points are defined as *PatchPoint1* to *PatchPoint7* for the patch, and *GndPoint1* to *GndPoint6* are the spline points for the ground plane. The total length of the shorting line includes *ShortL1* and *ShortL2*. A stub arm at the shorting line located at *ShortstubP* with the width and length defined as *ShortStubW* and *ShortStubL,* respectively. A radiating proximity coupling patch positioned at (*Patch2offsetX*, *Patch2offsetY)* is with the width and length of the patch set as *Patch2W* and *Patch2L*. The formable RG402 coaxial cable feeds the center of the board with its braid soldered to the ground plane at the bottom layer and its center core soldered at the top layer that feeds the microstrip line. Table 4 summarized the proposed design parameters.

### 2.4. The Low PIM Construction Consideration

A low PIM antenna design needs significant detail consideration to identify and avoid possible PIM sources like the non-linear material or junction. The proposed antenna follows explicit rules with similar construction considerations in [13] to achieve low PIM level below −150 dBc.

Ferromagnetic (e.g., nickel, iron, cobalt, etc.), ferrimagnetic (ferrites), and dielectric material (e.g., alumina 99.7% [33], etc.) can contribute to non-linearity when used as a base material, support or finishing. Any loose galvanic contact, dirt, dust, contaminated surfaces with metal flakes, moisture or oxidation, rough surface finishing, etc. are considered as nonlinear junctions. By understanding the collective of non-linearities of each PIM source, the proposed design details the consideration in material or component selection, component fabrication quality, housing design, radiating element trace, and radiating element/cable assembly process.

**Figure 11:**
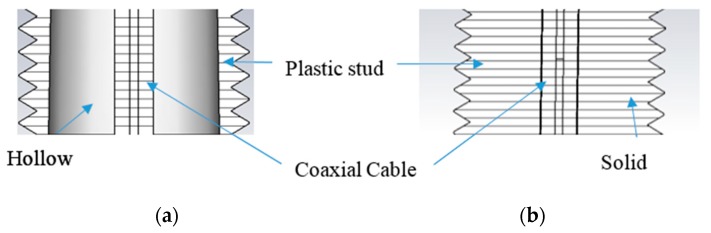
The plastic stud (**a**) with a hollow structure and (**b**) with solid ribs to hold the coaxial cable tightly.

The proposed design selects the low PIM-rated substrate laminate AD300 C with a reverse treated copper electro-deposited (ED) foil or in short as a reverse treated foil (RTF). RTF copper foil offers low profile tooth copper (smoother) in the inner layer of the copper to the substrate which has the most significant influence on PIM performance as studied in [34]. Also, the experimental study of PCB laminate material properties such as the dissipation factor and the moisture absorption has exhibit effect to the PIM level [34]. Article in [35] provides a general guide for choosing circuit materials for low PIM PCB antenna. As AD300C is a soft material based on woven fibreglass-reinforced Teflon, the solder joint of the coaxial cable can be the source of inconsistent PIM performance when the cable is prone to movement or microcracking. Therefore, careful design of the plastic housing by introducing minimal plastic recess at the cable exit and long ribs of the plastic stub to hold the coaxial cable tightly to minimize movement of cable and substrate is essentially important as shown in Figure 11.

In terms of PCB fabrication, the immersion tin finishing of the copper trace is used for soldering pad while the solder mask is suitable for non-soldering trace. Right PCB fabricator ensures more precision and additional cleaning process to produce smoother strip edges, less fracturing and contamination for low distributed PIM generations. A careful study of the solder pad design and dimension of the trace can yield quick soldering and ease of consistent wetting.

The proposed design includes pigtail cable, which consists of tin-soaked cable formable RG402 and low PIM rated connector, e.g., N and 4.3–10 connector. In preparation for the cable assembly, the cable needs a clean cut of striping with minimal burr. Besides, the joint between the connector and the cable required the right soldering equipment such as induction or resistant soldering. All the cable assemblies need to be tested with higher requirements before assembled to the antenna.

The proposed antenna has two galvanic joints that are the joint between the center core and braid of the cable to the PCB microstrip line pad and ground plane, respectively. It is noteworthy that using a barrel as the interface between the braid and the PCB generally to have consistent joint may paradoxically lead to a higher risk of soldering wetting problems. 

Besides, the radome housing design has a snap feature and also heat staking that houses the PCB between the top plastic radome and baseplate without any metal fastener. It facilitates the assembly and holds the PCB and cable firmly.

### 2.5. Simulated Radiation Pattern Comparison with Typical 2D Planar Discone Antenna

For simulation, the antenna model consists of the complete components inclusive of polycarbonate plastic radome and cable. The simulation model in CST Microwave Studio includes the actual 350 mm length of the cable similar to the prototype. Due to the significant structure size (inclusive the foamable RG402 coaxial cable) and super wideband, the antenna model needs to be simulated with a graphics processing unit (GPU) and in moderate accuracy in consideration of the computing load capability. Without the cable in the model may results in a high discrepancy in the frequency range 350–960 MHz as there is no designated wideband balun in the proposed design. Besides, any balun/choke for this antenna will be a substantial structure and not desired due to the size, cost, additional parts to build and a higher risk of PIM generation. 

**Figure 12:**
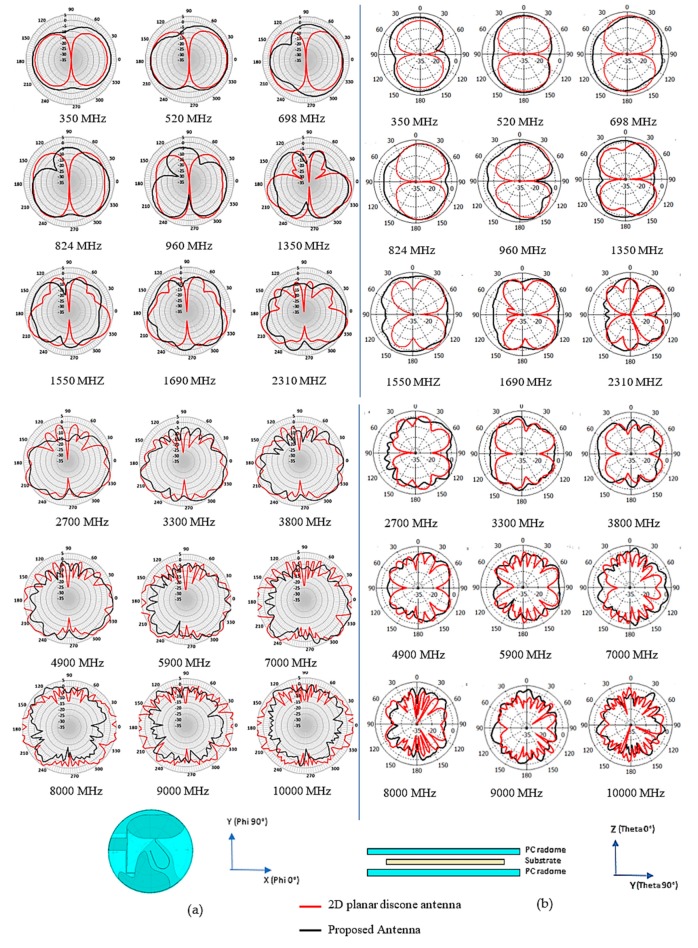
Comparison of the simulated radiation pattern of the conventional 2D planar discone antenna and the proposed antenna at (**a**) Azimuth Plane (X-Y Plane) and (**b**) Elevation 90° Plane (Y-Z Plane).

The dipole/monopole antenna that lies horizontally inherits the intrinsic deep azimuth null radiation pattern may lead to inconsistent and blind-spot coverage in the network. The proposed design offers some azimuth null reduction radiation pattern at some frequency range. Figure 12 shows the comparison of the simulated azimuth plane radiation pattern of the conventional 2D planar discone antenna and the proposed design. As mentioned before, the horizontal oriented 2D planar discone antenna has deep nulls at angle phi = 0°and phi = 180°.

In Figure 12a, the azimuth radiation pattern at 350 MHz–824 MHz shows improvement for a null reduction. However, the figure shows an additional null at 960 MHz. The radiation pattern also shows null reduction improvement from 1350 MHz to 7000 MHz. The overall null reduction at theta 90° plane cut may be a functional differentiation of performance for this type of antenna. The elevation 90° plane radiation pattern shows the null reduction at theta 90°, as shown in Figure 12b.

### 2.6. Antenna Performance Impact by Pigtail Cable Length 

In the field, the pigtail cable length is typically about 350 mm, and it is connected to a jumper cable above the ceiling. This results in the extension of the cable length of the antenna in operation. Therefore, it is essential to understand the unbalanced cable effect of an unbalanced electrically small antenna and make sure that the impact on the VSWR and radiation pattern with different jumper cable length is minimal. 

**Figure 13:**
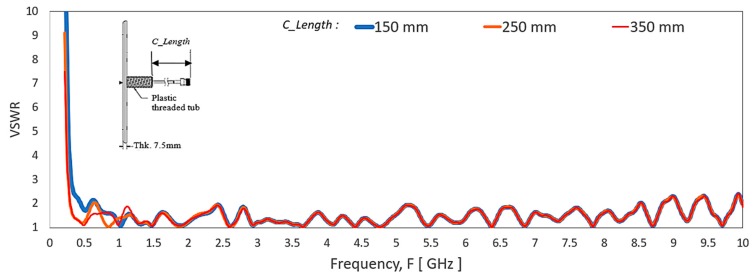
Simulated VSWR of the antenna with varying of cable length, *C_Length*.

As noted, the unbalanced cable effect will lead to some variance of performance of different pigtail cable length, *C_length*. Therefore, the exact cable length model in the simulation tool offers a more accurate comparison result of the antenna measurement. A few antenna cable lengths, *C_length* are simulated to investigate their impact on the VSWR and radiation pattern of the antenna. Figure 13 shows simulated VSWR with a varying cable length of the antenna. The simulated result shows a more significant impact at the frequency range of 350–760 MHz for the varying *C_length.* The effect of the cable length at the frequency range of 760 to 10,000 MHz is minimal. If the simulation model is with different length, there will be a large discrepancy in the simulated and measured results as a referenced result.

**Figure 14:**
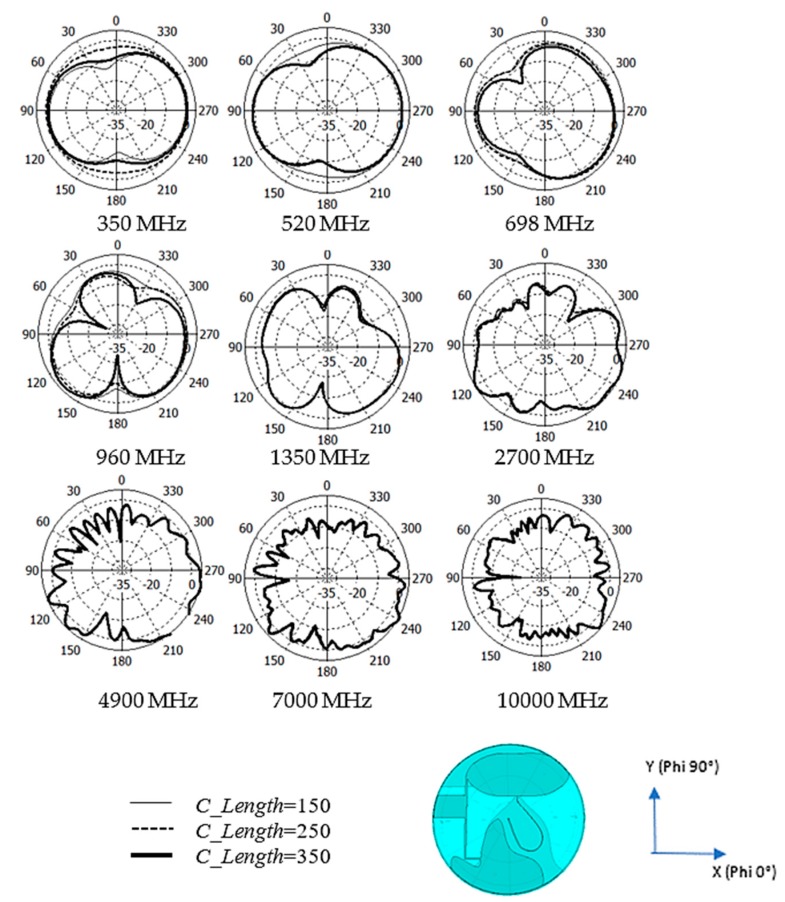
Comparison of simulated radiation pattern proposed dipole antenna at Azimuth Plane (theta 90°/X-Y) from 350 MHz to 10,000 MHz with a variance of cable length, *C_Length*.

During the VSWR measurement, the test cable is general coaxial cable type without armour or choke. The fluctuation of the VSWR curve caused by hand touching the test cable at the various location can determine qualitatively the severity of the unbalance cable effect. Because of the VSWR curve fluctuate under 2:1; hence the unbalanced cable is not critical for the application. Figure 14 shows the simulated antenna radiation pattern of varying *C_length* of the antenna. The simulated result exhibits variance in radiation pattern from 350–960 MHz. There is minimal radiation pattern variance for 1350–10000 MHz.

### 2.7. Simulated Parametric Studies on the Effect of Vvarious Structure Parameter Modification

**Figure 15:**
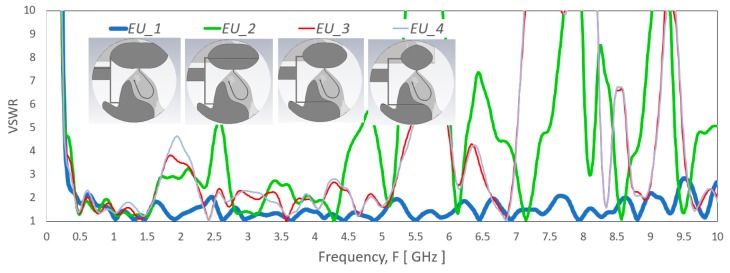
VSWR of the antenna with a different set of spline points (*EU_1*, *EU_2, EU_3* and *EU_4*) that construct the monopole elliptical-liked radiator patch. *PatchPoints1* to *PatchPoints7* defines the set of spline points in (*EU_1*, *EU_2, EU_3* and *EU_4*) that related to *PatchL.*

**Figure 16:**
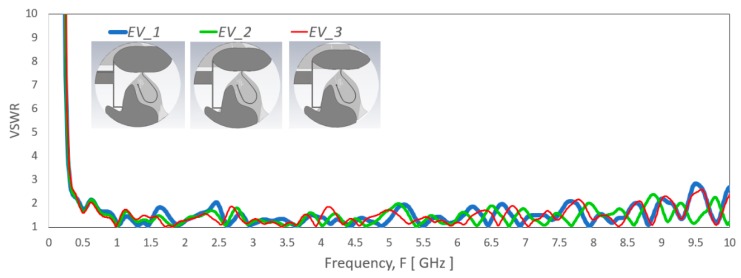
VSWR of the antenna with a different set of spline points (*EV_1*, *EV_2* and *EV_3* that construct the monopole elliptical-liked radiator patch. *PatchPoints1* to *PatchPoints7* defines the set of spline points in (*EV_1*, *EV_2* and *EV_3*) that related to *PatchW*.

The parametric studies on the effect of various parameter modification are presented based on the simulation result. Figure 15 shows VSWR effect by a different set of spline points (*EU_1*, *EU_2, EU_3* and *EU_4*). The simulated result shows significant mismatch, especially for the frequency range above 800 MHz when the monopole patch length, *PatchL* size reduced. Figure 16 shows the VSWR of the antenna related to the monopole patch width *PatchW* that defined by the set of spline points (*EV_1*, *EV_2* and *EV_3*). The VSWR vary significantly but still in matched level across the operating frequency range. The curvature near the feed has minimal change that maintains good match for the high band range, and the lowest frequency has expected little variations due to the proximity coupling patch at the top continue the longest electrical path.

**Figure 17:**
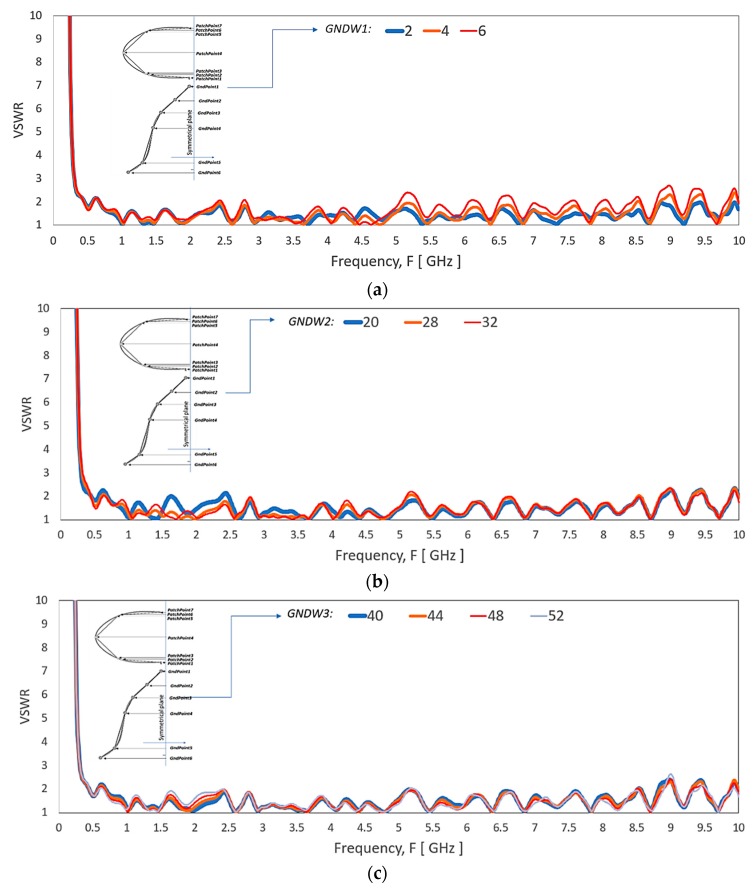
VSWR of the antenna by varying the (**a**) *GNDW1*, (**b**) *GNDW2* (**c**) *GNDW3* of the ground plane.

Figure 17 shows the spline points of bell-shaped ground plane shape effect on the VSWR level. The spline point nearer to the antenna feed has a significant impact on the upper-frequency range, as shown in Figure 17a. As the points are away from the feed, the effect of the points are more substantial to the lower frequency, as shown in Figure 17b,c.

**Figure 18:**
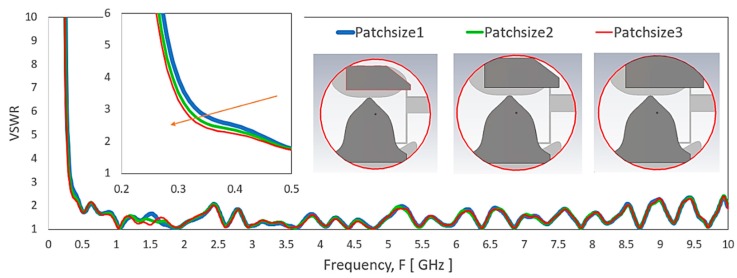
VSWR of the antenna by varying the proximity coupling patch to the monopole patch.

Figure 18 shows the VSWR of the antenna by varying the proximity coupling patch size. As the patch size increases with its patch above the bottom edge of the elliptical-liked shape, the VSWR shows minimal changes across the operating frequency except for a slow improvement for the lowest frequency range. 

**Figure 19:**
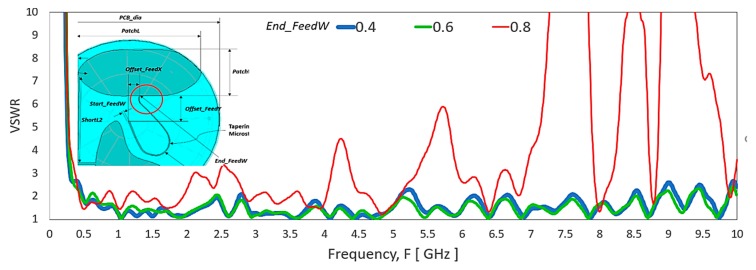
VSWR of the antenna by changing the width of the microstrip line, *End_FeedW*.

Figure 19 shows the width of the microstrip line, *End_FeedW* at the monopole patch has a substantial effect with a tiny change from 0.6 to 0.8 mm. There are multiple VSWR spikes and high VSWR level at multiple frequency ranges with the *End_FeedW* of 0.8 mm.

**Figure 20:**
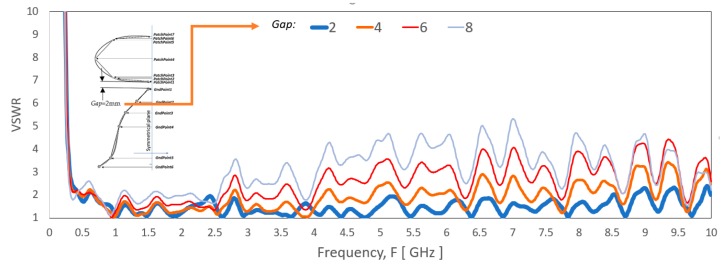
VSWR of the antenna with a few different gap, *Gap* between the elliptical-liked shape radiating element and the bell-liked shape ground plane.

Figure 20 shows the impact of the gap, *Gap* between the monopole patch and the ground plane on the VSWR of the antenna. The result shows that the impact is significant above 1 GHz. When the gap difference becomes more significant, the higher frequency range becomes mismatched. Nevertheless, the *Gap* is still vital at the lowest frequency range despite its slower change of VSWR. 

**Figure 21:**
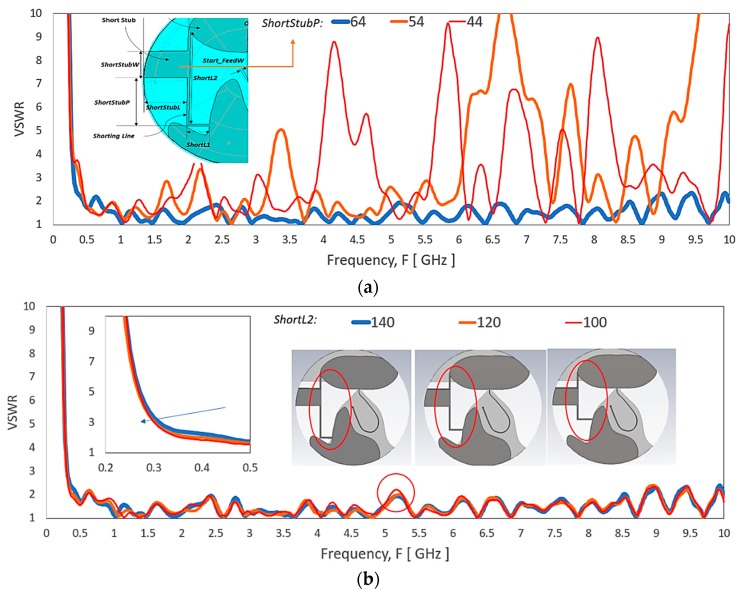
VSWR of the antenna by varying the (**a**) stub location, *ShortStubP* at the shorting between the monopole patch and the proximity ground coupling patch, and (**b**) shorting path length, *ShortL2*.

In Figure 21a, when the stub position at the shorting path, *shortstubP* decreases, the VSWR level rises from 1 GHz above. Figure 21b shows the VSWR of the antenna by varying shorting path length, *ShortL2*. Shorting the path length does not show a significant effect across the frequency range with a slow and slight improvement at 350 MHz range in the expense of a VSWR spike at 5.25 GHz.

## 3. Fabrication and Measurement Result

**Figure 22:**
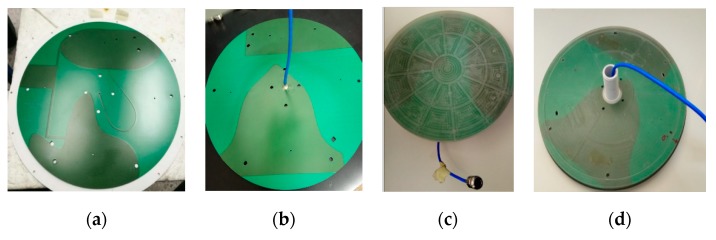
The photo of PCB with green mask (**a**) Top View and (**b**) Back View. The picture of the prototype with a transparent radome and cable assembly (**c**) Top View and (**d**) Back View.

Figure 22 shows the photos of the proposed antenna PCB prototype. It is worth noting that a prototype fabricated with decent production quality is vital to verify the best measurable and repeatable PIM performance. In-house PCB rapid prototyping facilities that use milling technology may result in doubtful smoothness of mechanical etching to validate the feasibility of a low PIM antenna prototype level. A set of CNC milling machined transparent PC radome housing prototype was fabricated to house the PCB antenna. A formable coaxial cable based on RG402 that assembled with a low PIM N connector is soldered to the ground of the PCB. 

**Figure 23:**
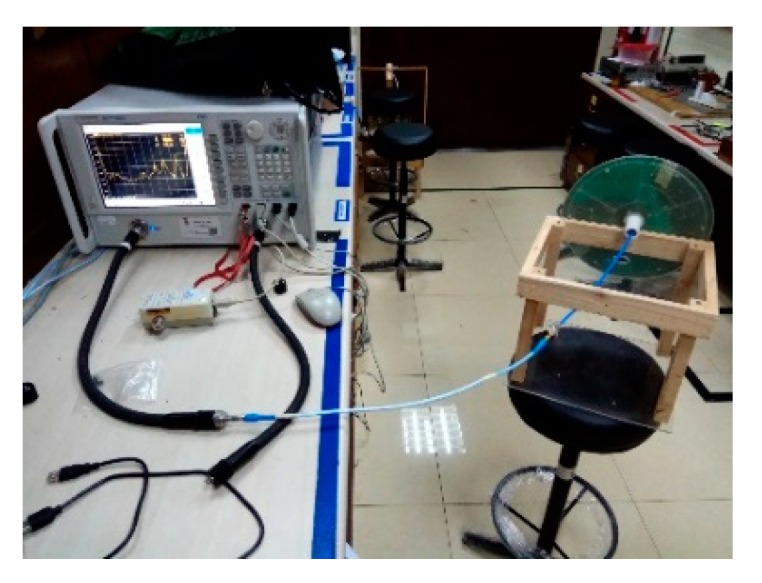
Photo of the VSWR measurement setup using the N5227A PNA Microwave Network Analyzer.

**Figure 24:**
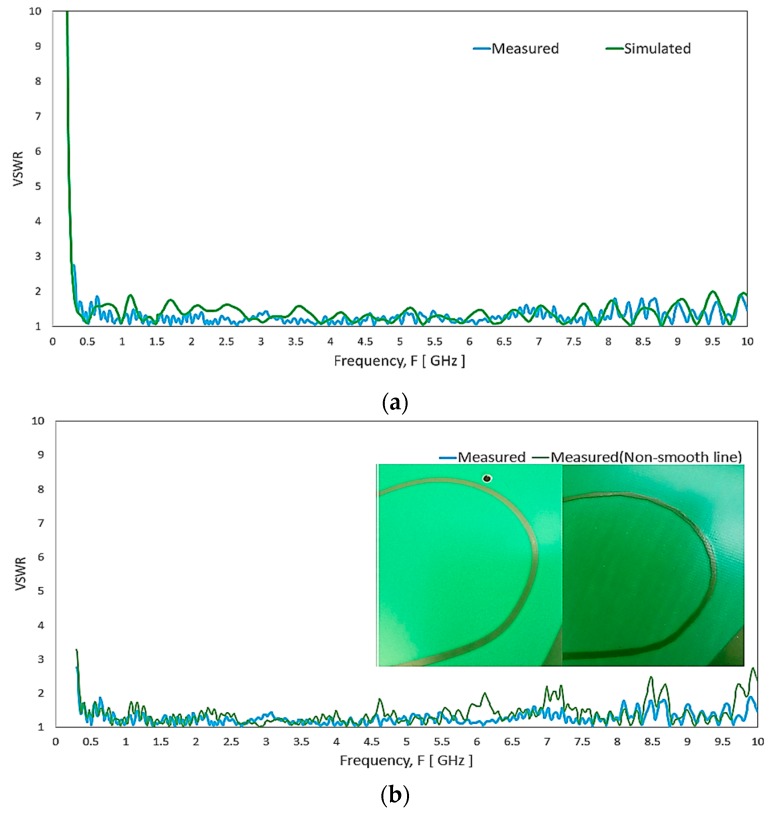
(**a**) Comparison of the simulated and measured VSWR of the antenna. (**b**) The measured VSWR of the antenna with the segmented edge and smooth edge microstrip line.

Figure 23 shows the VSWR measurement setup using an N5227A PNA Microwave Network Analyzer. Figure 24a depicts the measured and simulated VSWR of the proposed antenna. The simulated result shows the antenna is covering from 350 to 9450 MHz for VSWR < 2:1. However, the measured result shows that the antenna can cover a broader range of 350 to 10,000 MHz with a substantial band ratio of 28.57:1. The discrepancy can be caused by insufficient mesh resolution to represent the smooth edge of the fine curvature arc microstrip line in the simulation model. Figure 24b exhibits the spike of VSWR can occur for segmented edge instead of a smooth edge microstrip line of the antenna. Nevertheless, the higher frequency range 6000–10,00 MHz may not be the focus for the current DAS system but possible future expansion.

**Figure 25:**
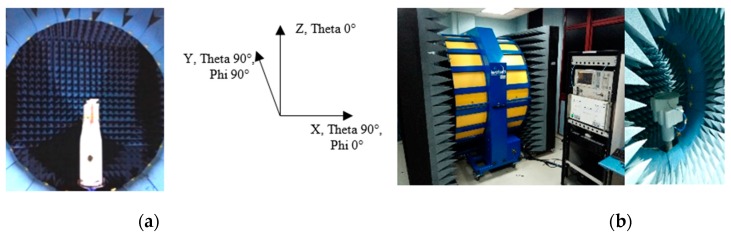
Measurement setup in (**a**) Satimo SG24 (350–6000 MHz measurement) and (**b**) Satimo Starlab (7000–10,000 MHz) for the radiation pattern measurement of the antenna.

**Figure 26:**
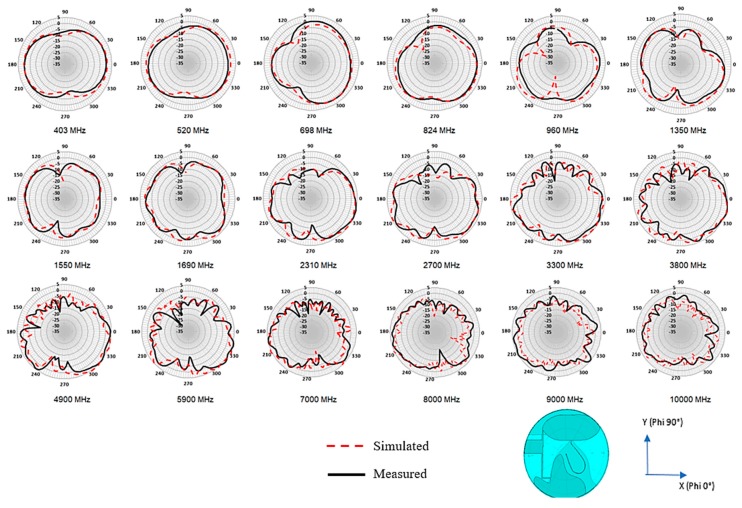
Simulated and measured azimuth (theta 90°/ X-Y) plane radiation pattern of the proposed antenna from 350 MHz to 10,000 MHz.

Figure 25 shows the Satimo SG24 and Satimo Starlab measurement system setup that is used to measure the radiation pattern of the proposed antenna for the frequency range 400–6000 MHz and range 6000–10,000 MHz, respectively. Figure 26 shows the measured and simulated radiation pattern at various frequency point for the horizontal plane (azimuth/theta 90° plane). For the sake of brevity, the elevation plane radiation pattern is not presented. The radiation pattern result shows a good correlation between the simulated result and the measured result. From 520 to 960 MHz, the measurement result shows a better null reduction compared to simulated results. As the frequency increases, the ripple is observed on the radiation pattern. The ripple can be the effect of spurious radiation from the cable and higher modes excitation in the travelling wave across the larger electrical aperture when the frequency increases. Despite all that, it has a better performance compared to a typical 2D discone-type antenna.

**Figure 27:**
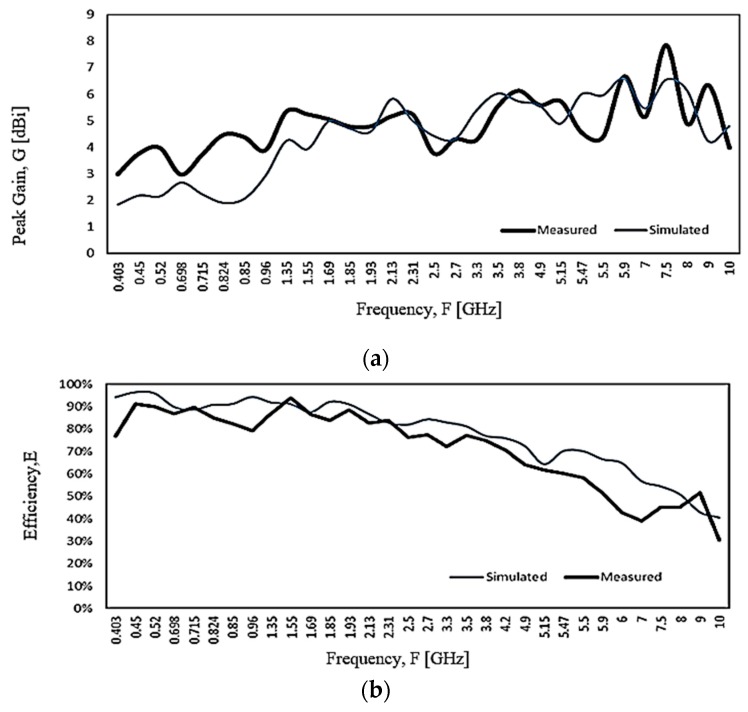
The proposed antenna measured and simulated (**a**) peak gain and (**b**) efficiency.

Figure 27a shows a comparison between measured and simulated antenna peak gain within the operating frequency range. The simulation gain result from 600 MHz onwards conforms well with the measurement result. It has a higher measured gain at the 350 MHz band. The discrepancy can be the result of the higher uncertainty of the measurement chamber SG 24, which is more suitable for the measurement above 400 MHz and ripple of the radiation pattern caused by unbalanced cable effect. Figure 27b shows a comparison between the measured and simulated efficiency of the proposed antenna. Both measured and simulated results correlated to each other with minimal variation. The trend of the total efficiency shows the efficiency drop with the frequency, from about 85–90% at the UHF band to about 30% at 10 GHz.

**Figure 28:**
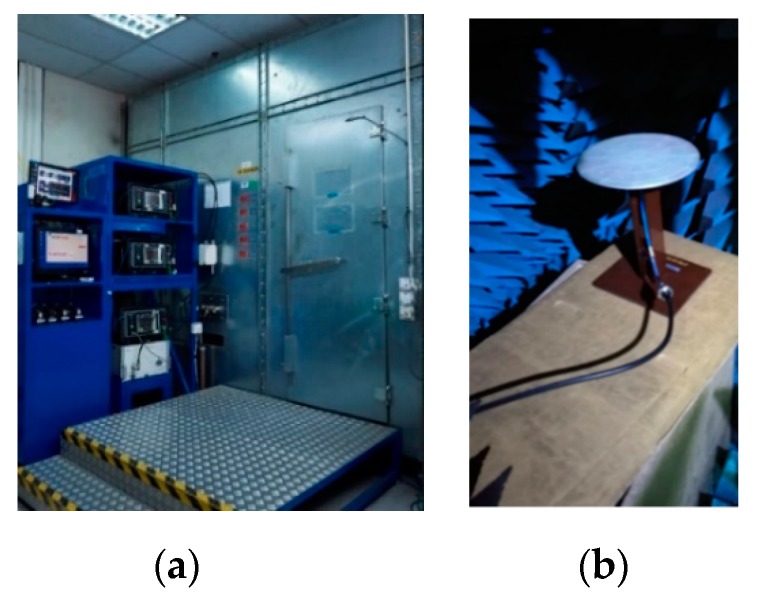
(**a**) The PIM measurement system with both the PIM Analyzer and PIM chamber. (**b**) The antenna under test inside the PIM chamber.

Figure 28 shows the PIM measurement setup with the PIM Analyzers and the 10 feet × 10 feet × 10 feet anechoic PIM chamber. The antenna third-order PIM level was measured with excited two tones of 43 dBm frequency carrier inside the PIM chamber. Commercial PIM Analyzers (AWT PS2M40, Kaelus iQA-700 HC, and Kaelus iQA-1920C) are used, respectively, to measure the PIM level at the TETRA 400 MHz Band, 700 MHz band and 1920 MHz band 

The proposed antenna was tested for three frequency bands: 380, 700 and 1920 MHz. The measurement uses the reverse measurement mode, in which the PIM level is measured in the reverse direction from the test cable. The PIM analyzer is set in the frequency sweep mode to measure a range of frequency band. In frequency mode, the analyzer excites the two-tone carriers and measures the reverse PIM level for 7 s. This duration will enable sufficient time to observe if a consistent result is achievable. If the design has been verified with good results consistently, the design is considered to be low PIM rated. Hence, both frequency and time are essential to detect inconsistency. A lot of factors like the process of soldering, cable assembly, the mating of the connector during testing can cause inconsistent results. Before any measurement, the connector of the antenna is tightened according to the designated connector torque, e.g., N connector uses the torque wrench setting of 2.8 Nm for consistent measurement.

Table 5 shows the transmitted and received frequency range of the available PIM Analyzer equipment. For the 380 MHz bands, the two-tone carriers are sweeping between 390–400 MHz, and the measured third-order PIM level is performed at 380–384 MHz. For the 700 MHz band, the two-tone carriers sweep between 728–757 MHz range, and the measured third-order PIM level is shown at 776–786 MHz range. For the 1920 MHz band, the two-tone carriers sweep between 1930–1990 MHz, and the third-order PIM level is measured at the 1870–1910 MHz range. The DAS applications specified the requirement of the peak third-order PIM level < −150 dBc.

**Figure 29:**
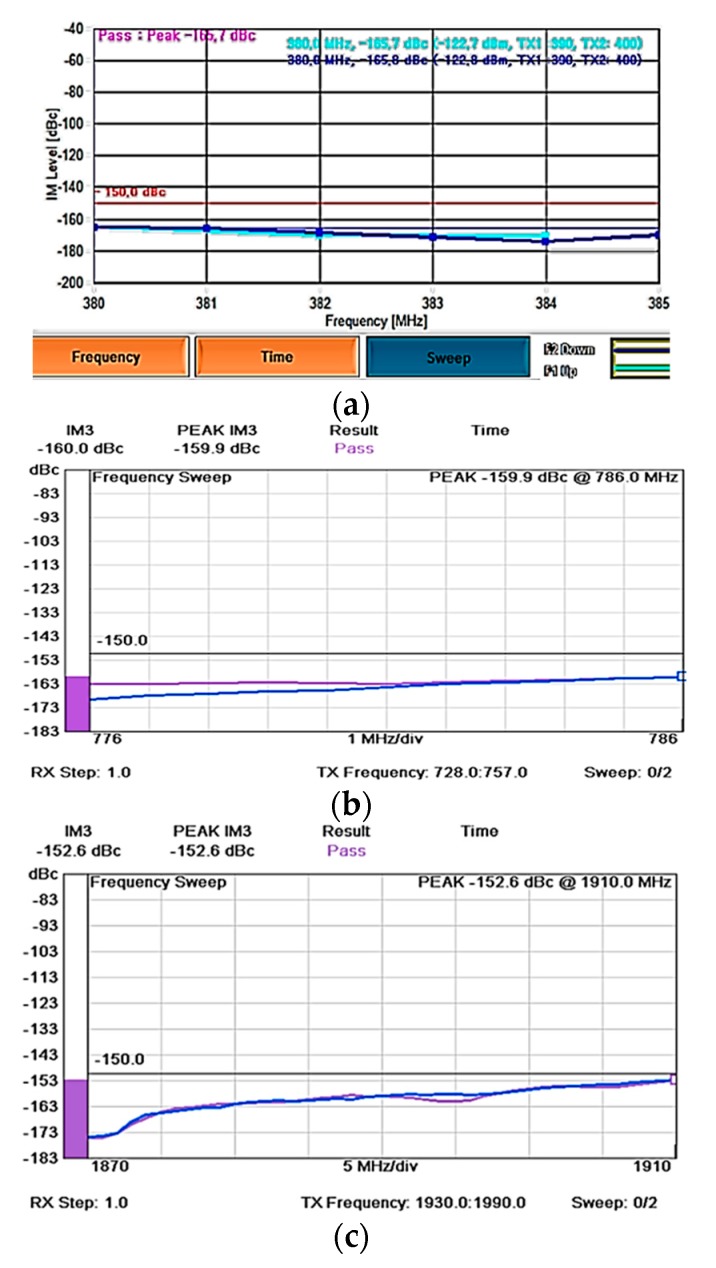
The measured PIM level for **(a)** 380 MHz-band **(b)** 700 MHz-band and **(c)** 1920 MHz-band.

Figure 29 shows the measured PIM level of the three-representative frequency range (380 MHz-band, 700 MHz-band, and 1920 MHz-band). The measured peak PIM level is −165.7 dBc, −159.9 dBc, and −152.6 dBc for 380 MHz-band, 700 MHz-band, and 1920 MHz-band, respectively.

Table 6 summarizes the brief comparison between other designs and the proposed antenna. An array of radiating elements [17,18,19,20,21,22] can achieve good horizontal polarized omnidirectionality radiation pattern but can be extremely large if to support down to 350 MHz and limited bandwidth. 

Conventional single dipoles or monopoles [23,24,25,26,27,28,29,30] offer smaller size radiating elements but not small enough for 350 MHz. Besides, such radiating elements suffer from deep null azimuth radiation pattern when lied horizontally. Some of the reported works do not consider the design with pigtail configuration, and the cable effect is not considered except in [13,31]. Hence, the radiation pattern result may have a higher discrepancy as no cable is modelled in their simulation. Also, these antennas do not include any balun design. The proposed design offers simple and has super wideband with improved radiation pattern from deep null in most of the frequency ranges. The proposed design has a better VSWR (e.g., VSWR typically better than 1.8:1) performance that can harmonize the detuning effect due to dielectric loading, etc. The diameter of the proposed design 270 mm is 0.32*λ*_L_ compared to 342.9 mm (0.43 *λ_L_*) of resized conventional 2D discone antenna. In characterizing the antenna operating bandwidth, the fractional bandwidth, *FBW_v_ (ω_0_)* is calculated based on the expression given below as below:

(1) $$FBW_{v}\left(\omega_{0}\right)=\frac{\omega_{-}-\omega_{+}}{\omega_{0}}$$

where *ω_+_* and *ω_−_* are the frequencies above and below *ω*_0_, respectively, where their VSWR is equal to *s*. Matched VSWR bandwidth and *Q_VSWR_* are inversely related as below [36,37]:

(2) $$Q_{VSWR}\approx \frac{2\sqrt{\beta }}{{\mathrm{FBW}}_{v}},\;\sqrt{\beta }=\frac{S-1}{2\sqrt{S}}\leq 1$$

The lower bound Q*_lb_* as a function of the antenna is given by [38]:

(3) $$Q_{lb}=\eta \left(\frac{1}{{\left(ka\right)}^{3}}+\frac{1}{ka}\right)$$

The fractional bandwidth, FBW_v_ for the proposed modified antenna and conventional 2D discone antenna (as in Figure 1) are 186.47% and 178.29% respectively. The Q-factor (*Q_VSWR_*, *Q_Lb_*) for the proposed design are respectively (0.379, 2.040) and conventional 2D discone antenna (0.397,1.235).

The proposed design shows lower Q_VSWR_ with a smaller diameter even with a higher *Q_Lb._*

## 4. Antenna VSWR and Radiation Pattern Impact on the Ceiling Tiles with/out Metal Frame 

**Figure 30:**
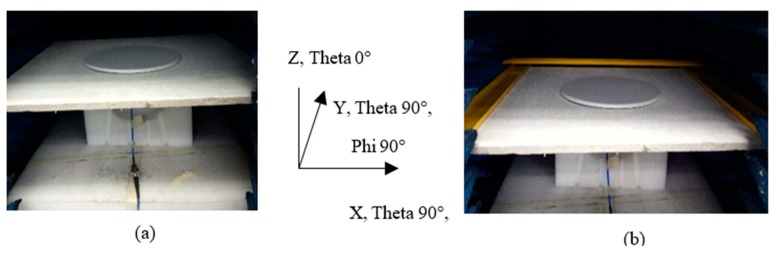
Photo of the proposed antenna on the top of acoustic ceiling tiles (**a**) without and (**b**) with half of the metal frame.

The application of the proposed antenna limits to mount on the (mineral and fiber)-filled acoustic ceiling due to its asymmetrical dipole characteristics. It will have a significant detuning effect if to install on any metal ceiling or metal-backed ceiling. Typical 600 mm × 1200 mm × 15 mm ceiling tile size is too large for the available measurement chamber; therefore, only a half size (600 mm × 600 mm × 15 mm standard acoustic tile) is used to verify its effect to antenna performance. Other than the ceiling tile material effect, three sides of the edge metal frame that represent half of the typical tile installation was measured as well. Figure 30 shows a photo of the antenna taken with the acoustic tiles with or without the frame.

**Figure 31:**
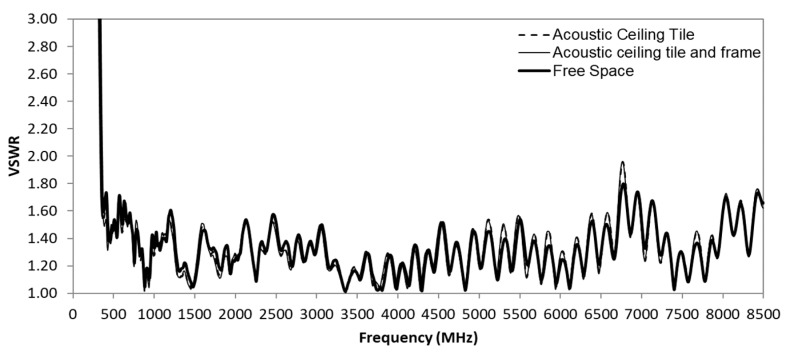
Ceiling tiles and frame effect to the antenna measured VSWR.

**Figure 32:**
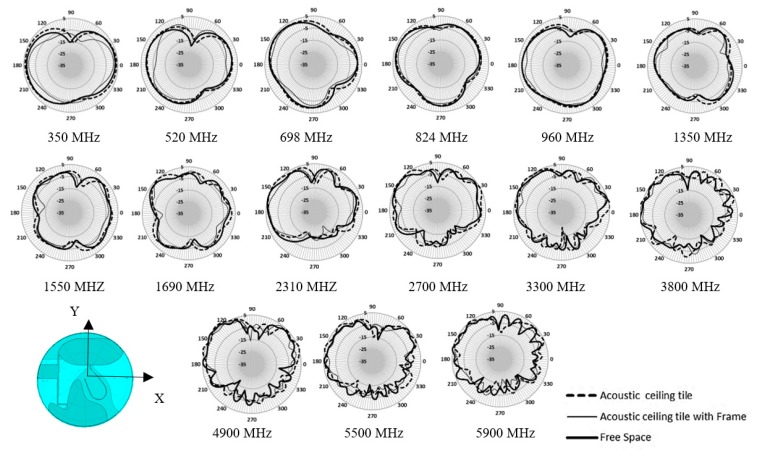
Ceiling tiles and frame effect to the antenna measured azimuth plane (X-Y plane) radiation pattern.

Figure 31 shows the comparison of measured antenna VSWR in free space, on the acoustic ceiling tile with and without the metal frames. All these three conditions show minimal impact on its VSWR. There is further investigation of the radiation pattern effect, as shown in Figure 32. The radiation pattern measurement shows the ceiling material causes little variance. The PCB antenna is designed with its PCB slightly elevated from the ceiling material with ribs. This slight elevation helps in minimizing the impact of ceiling material on the antenna. However, those different densities, thickness, material, and hardness of ceiling tiles may still impact the performance of the antenna. In Figure 32, we can observe a more variance in the radiation pattern with the metal frame at the edge of the ceiling tile. The difference is more evident at the lower frequency range from 350 MHz to 520 MHz. 

## 5. Conclusions

A novel design ULP, low PIM, and super wideband ceiling mount in-building antenna for both cellular and PS/PPDR DAS was presented. The proposed antenna has a superwide bandwidth with band ratio of 28.57:1 covering a frequency range of 350–10,000 MHz in a small disc-shaped radome with a diameter of 270 mm. The design utilizes the super wideband characteristics of the conventional 2D discone antenna and combines features to miniaturize the antenna into the disc-shaped radome. The features include the introduction of shorting path between an elliptical-liked monopole and bell-shaped ground plane, a stub at shorting line, offset structure both x-z and y-x plane and a proximity coupling patch to maximize electrical path to support the lowest frequency 350 MHz range. The proposed antenna reduces the antenna size to about 62% compared to the 342.9 mm diameter radome. These modification steps also result in the null reduction of the azimuth radiation pattern in most of the operating frequency range. 

The antenna design has considered the detail of material selection, processes and dimensions of the trace to meet the industry low PIM performance requirement of <−150 dBc. A proper low PIM prototype is fabricated to verify the performance. Both measured VSWR and radiation pattern conformed well to the simulated results. The measured third-order PIM peak levels for 350 MHz, 700 MHz, and 1920 MHz bands are < −150 dBc (2 × 43 dBm). Also, the ceiling tiles with and without metal frame effect on the antenna performance were investigated.

## 6. Patents

A patent [39] of this design concept is in the application phase.

## Figures and Tables

**Table 1:** Commercial antenna product standard sizes for PS/PPDR and the LTE cellular band.

Ref.	Antenna Size (mm)	VSWR Specification (MHz)	Remarks
[8]	298 Dia. × 152	<3.0:1 @ 380–520 <2.0:1 @ 600–6000	Standard Height
[9]	248 Dia. × 150	<1.9:1 @ 350–470 <1.8:1 @ 617–960 <1.9:1 @ 1710–6000	Standard Height
[10]	288 Dia. × 136	<2.5: 1 @ 380–520 <2.0:1 @ 698–6000	Lower Profile but a special shape
[11]	298 Dia. × 133	<3.0: 1 @ 380–520 <2.0:1 @ 600–6000	Lower Profile compared standard
[12]	335 Dia. × 124	<1.7:1 @ 380–520 <1.5:1 @ 617–790 <2.0:1 @ 790–960 <1.9:1 @ 1695–2200 <2.2:1 @ 2700–6000	Lower Profile compared standard

Notes: (1) Some frequency ranges simplified into a broader range for rather than following application band. (2) Cellular includes commercial LTE bands, e.g., 600–960 MHz, 1350–1550 MHz, 1690–2700 MHz, 3300–4200 MHz, and 4900–5900 MHz. (3) PPDR/PS lowest UHF band include 350–520 MHz Band.

**Table 2:** Commercial ULP\low profile DAS antenna products that for converged PS\PPDR and LTE Cellular.

Ref. No.	Antenna Size (mm)	VSWR Specification (MHz)	Remarks
[20]	265 × 182 ×15	<2.5:1 @ 380–470 <1.8:1 @ 1427–2700	Rectangular shape
[21]	311 Dia. × 9.5	<2.0:1 @ 380–570 <2.0:1 @ 698–960	Round shapebut the cellular band covering only the typical low LTE band
[22]	309 × 229 × 21.5	<2.2:1 @ 380–3800	Rectangle shape
Proposed Design	270 Dia. × 7.5	<2.0:1 @350–1000	Round shape

**Table 3:** The summary of the ULP ceiling mount antenna requirement that enables PS\PPDR (UHF 350 MHz Band) and cellular DAS application.

Characteristic	Parameter Value	Remarks
**Frequency range**	350–6000 MHz	350–370 MHz is additional frequency bands for some country/ region needs (e.g., China) for PPDR **
**VSWR**	<2:1@350–6000 MHz	Super wideband frequency range
**Radiation Pattern**	Omnidirectional in Horizon	At least it has reduced null at azimuth plane if pure omnidirectionality not achievable.
**Form factor**	Circle disc-shaped with a plastic threaded stud for mount application	A radome is necessary for protecting the antenna from dust and protect the feeding cable joint for reliable low PIM performance. Typical industry interpretation of circle shape as an omnidirectional antenna while rectangle as a directional antenna
**Connection**	Pigtail coaxial cable	Low PIM coaxial cable assembled with N or 4.3–10 connector that exit at the center of the radome
**PIM (2 × 43 dBm)**	<−150 dBc	Measured with two tones carrier with 43 dBm at various frequency band.

** PPDR is public protection and disaster relief (for radiocommunication systems [32]).

**Table 4:** Proposed antenna parameters.

Antenna Parameter	Value (mm)	Antenna Parameter	Value (mm)
*PCB_dia.*	264.6	*ShortL1*	29.01
*Offset_FeedX*	14.97	*ShortL2*	140.14
*Offset_FeedY*	40.80	*ShortW*	55.52
*Feedline_L*	195.33	*ShortStubL*	40.00
*Start_FeedW*	1.98	*ShortStubP*	74.00
*End_FeedW*	0.60	*PatchPoint1*	(−0.7, 0)
*FeedLength*	199.75	*PatchPoint2*	(−55, 4)
*PatchL*	179.76	*PatchPoint3*	(−60, 6)
*PatchW*	75.00	*PatchPoint4*	(−90, 37.5)
*Patch2L*	189.91	*PatchPoint5*	(−60, 69)
*Patch2W*	74.30	*PatchPoint6*	(−55, 71)
*Patch2_offsetY*	58	*PatchPoint7*	(−0.7, 75)
*Patch2_offsetX*	71	*GndPoint1*	(−4, −2)
*Proxi_Peri*	188	*GndPoint2*	(−24, −23)
*GndL1*	135	*GndPoint3*	(−44, −43)
*GndL2*	155	*GndPoint4*	(−55, −67)
*GndW1*	8	*GndPoint5*	(−70, −122)
*GndW2*	180	*GndPoint6*	(−90, −137)
*ExtX1, ExtY1*	(−97, −68)	*ExtX2, ExtY2*	(−100, −80)

**Table 5:** The transmit and receive frequency range of the PIM Tester.

PIM Tester Unit	Tx Frequency Range	Rx Frequency Range
AWT PS2M40	390–400 MHz	380–384 MHz
Kaelus iQA-700 HC	728–757 MHz	776–787 MHz
Kaelus iQA-1920 C	1930–1990 MHz/2110–2155 MHz	1710–1755MHz/1850–1910MHz

**Table 6:** Comparison summary of other works and the proposed design.

Design and Size	Structure Figure	Description	Frequency Range (Bandwidth/Band Ratio)	Other Remarks
[3] Size N\A Not ultralow profile	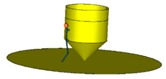	A conical radiating element mounted on the ground plane and a ground leg that capacitively coupled (using capacitor) to the conical radiating element extending from the ground plane.	0.38–6 GHz (176%/15.8:1)	Low profile type antenna for supporting the lowest frequency of 380 Mhz Omnidirectional Vertical Polarized The curvature and tapering feature provides a smooth transition widen the super-wide bandwidth. There is either a form of shorting or parasitic coupling from the ground plane to the monopole radiating element.
[4] 310 mm (Dia.) × 145 mm Radome & Radiating element height 139 mm	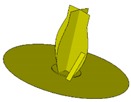	Two PCBs are placed perpendicularly as the top radiating element on top of a flat ground plane. The top radiating element is shorted using a stamped metal strip which is secured with fasteners	0.38–6 GHz (176%/15.8:1)	
[5,6] 298 mm (Dia.) × 133mm (H)	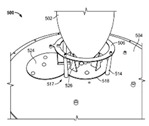	Consists of a conical radiating element, a shorted parasitic annular ring and a ground plane.	0.38–6 GHz (176%/15.8:1)	
[7] Size N/A	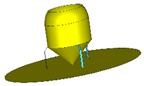	The aluminium radiating element is with the cup-type plum-shaped-liked cone structure. The radiating element has two shorting points with resistors, and one shorting leg to lower down the profile of the antenna.	350–960/1710–2700 MHz	
[14] 120 mm (Dia.) × 1.0 mm	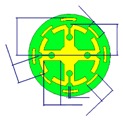	Four arc printed dipole array with four parasitic strips and four directors.	1.58–3.88 GHz (84.2%/2.45)	Planar type of radiating elements placed in a circular array to achieve an omnidirectional radiation pattern Horizontal Polarized Radiating element typically integrated with balun Limited bandwidth to support super wideband When the design is scaled to support 350 MHz, the size can be significantly large.
[15] 80mm (Dia.)	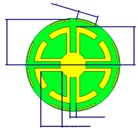	Four arc printed dipole array with a pair of parasitic strips for each dipole.	2.23–4.11 GHz (59.3%/1.84:1)	
[16] 100mm (Dia.)	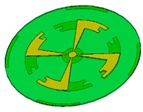	The antenna consists of four pairs of flag-shaped radiators, a balun for balance–unbalance transformation, and four parasitical strips for bandwidth enhancement.	1.76–2.68 GHz (41%/1.52:1)	
[17] 196 mm (Dia.) × 0.8128 mm [18] 132 mm (Dia.) × 1 mm [19] 176 mm (Dia.) × 1 mm	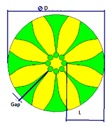	Tapered slots antenna (Vivaldi) in a circular array Tightly arranged Vivaldi element in circular array exhibits wider bandwidth and with smaller size.	1.9–2.7 GHz (34.78%/1.42:1) [17] 1.45–14.3GHz (163.17%/9.9) [18] 1.28–11.51 (159.97%/9:1) [19]	
[23] 226.8 mm × 453.6 mm × 0.127 mm	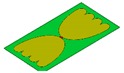	Bulbous concept dipole of the convex arm with circular-serrated edge	0.3–3 GHz (163%/10:1)	Symmetrical arm dipole antenna. Horizontal polarized A substantial size to support the lowest frequency of 350 MHz Dipole elevation liked radiation pattern at the azimuth plane when lied at the horizontal plane
[25] 230mm × 230mm × 30mm	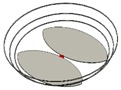	A bowtie antenna loaded with loops. It has a thickness of 30 mm so may not able to be classified as ULP	0.42–5.5 GHz (172%/13:1)	
[26] 90 mm × 135 mm × 1.575 mm	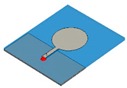	The asymmetrical dipole of circular shape (substrate *Ɛr* = 2.33) The feed is perpendicular to the antenna plane, but no inclusion of the cable effect in their model	0.7986–17.4663 GHz (182.5%/21.9:1)	Dipole elevation liked radiation pattern at the azimuth plane when lied at the horizontal plane. Simulated result in [30] shows deep null in the antenna design but not in [28] that shows non-correlation between both results. This discrepancy can be due to the unbalanced feed that has the cable effect. The cable effect will change the performance that it needs an accurate simulation model that includes the pig-tail cable or test cable effect.
[27] 140 mm × 107.3 mm × 1.524 mm	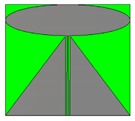	Elliptical monopole on trapezoid ground plane (Substrate *Ɛr* = 3.48)	0.4–9.51 GHz (184%/21.6:1)	
[28] 90 mm × 124 mm × 1.524 mm [30] 90 mm × 124 mm × 0.1 mm	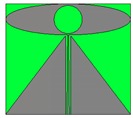	Elliptical monopole on trapezoid ground plane with a circular shaped cutout in the elliptical monopole patch (LCP Substrate *Ɛr* = 3.0)	0.44–10.6 GHz (184%/24.1:1) [28] 0.435–14.52 GHz (188.37%/33.4:1) [30]	
[29] 110 mm × 124 mm × 1.524 mm	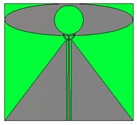	Elliptical monopole on trapezoid ground plane with a circular shaped cutout in the elliptical monopole patch. (LCP Ɛr=3.0, tan δ =0.002, thk.=0.1mm)	1.02–24.1 GHz (183.76%/23.61:1) [29]	
[13,31] 90 mm × 151 mm × 0.76 mm	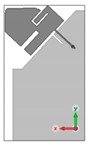	Offset Monopole, offset feeding point and slanted feeding edge ground plane. The monopole with multiple radiating arms. (AD300 Substrate *Ɛr*=2.97, thickness=0.76mm)	Dual wideband 0.698–0.96 GHz and 1.35–3.80 GHz (31.6% and 95.1%/5.4)	The reduced null radiation pattern when lies at the horizontal plane Low PIM construction Inclusive of cable effect in the simulation and measurement result. Substantial size to cover the lowest frequency of 350 MHz
Proposed Design 264.6mm (Dia.) × 0.76 mm	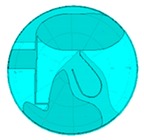	Elliptical liked shape monopole with bell-shaped ground plane that constructed with spline points for freedom of wideband matching. Fully offset radiating feeding point along the X-Z plane and Y-Z plane. The microstrip line is tapered and routed to the center of the antenna. Shorting path with stub (AD300 Substrate *Ɛr* = 2.97, thickness = 0.76mm)	0.35–10GHz (186%/ 28.57:1)	Super wideband Low PIM Azimuth reduced null radiation pattern when lies at the horizontal plane, especially important LTE band. Radiating element maximized its electrical length of a circular radome.

Notes: 1. The unbalance cable effect of a small antenna varies its performance significant with the cable length of the test cable or jumper cable in the field. Therefore, the proposed antenna includes the 350 mm pigtail cable in simulation model as close as the application as possible to provide a more accurate result. Any change of test cable or jumper cable length must not impact the VSWR performance too much. 2. Some of the figures are not in actual proportional dimensions or scaled, and are for illustration reference purposes only.
